# Plasma matrix metalloproteinase 1 improves the detection and survival prediction of esophageal squamous cell carcinoma

**DOI:** 10.1038/srep30057

**Published:** 2016-07-20

**Authors:** Yu-Kuei Chen, Chun-Wei Tung, Jui-Ying Lee, Yi-Chun Hung, Chien-Hung Lee, Shah-Hwa Chou, Hung-Shun Lin, Ming-Tsang Wu, I-Chen Wu

**Affiliations:** 1 Department of Food Science and Nutrition, Meiho University, Pingtung, Taiwan; 2 School of Pharmacy, Kaohsiung Medical University, Kaohsiung, Taiwan; 3 Division of Chest Surgery, Department of Surgery, Kaohsiung Medical University Hospital, Kaohsiung, Taiwan; 4 Research Center for Environmental Medicine, Kaohsiung Medical University, Kaohsiung, Taiwan; 5 Department of Public Health, Kaohsiung Medical University, Kaohsiung, Taiwan; 6 Department of Laboratory Medicine & Department of Research, Education & Training, Kaohsiung Municipal Hsiao-Kang Hospital, Kaohsiung Medical University, Kaohsiung, Taiwan; 7 Division of Gastroenterology, Department of Internal Medicine, Kaohsiung Medical University Hospital, Kaohsiung, Taiwan; 8 Faculty of Medicine, Department of Medicine, College of Medicine, Kaohsiung Medical University, Kaohsiung, Taiwan

## Abstract

This study aimed to identify noninvasive protein markers capable of detecting the presence and prognosis of esophageal squamous-cell carcinoma (ESCC). Analyzing microarray expression data collected from 17-pair ESCC specimens, we identified one protein, matrix metalloproteinase-1 (MMP1), as a possibly useful marker. Plasma MMP1 was then measured by enzyme-linked immunosorbent assay (ELISA) in 210 ESCC patients and 197 healthy controls. ESCC patients had higher mean levels of MMP1 than controls (8.7 ± 7.5 vs. 6.7 ± 4.9 ng/mL, p < 0.0001). Using the highest quartile level (9.67 ng/mL) as cut-off, we found a 9.0-fold risk of ESCC in those with higher plasma MMP1 after adjusting for covariates (95% confidence interval = 2.2, 36.0). Heavy smokers and heavy drinkers with higher plasma MMP1 had 61.4- and 31.0 times the risk, respectively, than non-users with lower MMP1. In the survival analysis, compared to those with MMP1 ≤ 9.67 ng/mL, ESCC patients with MMP1 > 9.67 ng/mL had a 48% increase in the risk of ESCC death (adjusted hazard ratio = 1.48; 95% CI = 1.04–2.10). In conclusion, plasma MMP1 may serve as a noninvasive marker of detecting the presence and predicting the survival of ESCC.

The incidence of esophageal cancer is increasing rapidly. In 2012, it was the eighth most common and the sixth most deadly cancer worldwide^1^. In the highest risk areas, such as China, Japan and Taiwan, more than 90% of the cases involve squamous cell carcinoma^2^. Because esophageal cancer is usually diagnosed in its late stages, the 5-year survival rate of this disease is reported to be only 15% to 25%^3^. Early diagnosis has so far been the only means of achieving better outcomes. Alcohol drinking and smoking are major risk factors for ESCC in most parts of the world^3^. In Taiwan, betel quid chewing is another important risk factor^4^,^5^. There is no consensus regarding the efficacy of endoscopic surveillance for ESCC, even among high-risk populations. In addition, no reliable diagnostic marker for ESCC has been found. The purpose of this study was to identify non-invasive protein markers to assist in the diagnosis of ESCC in clinical settings.

Studying previous established microarray data obtained from 17 paired esophageal tumor and adjacent normal tissues collected from 17 ESCC patients^6^, we identified the top seven genes associated with the most significant differences in expression between tumor and normal tissues. One of these, matrix metalloproteinase 1 (MMP1), is a well-known secreted protein associated with cancer^7^,^8^. MMPs are a family of zinc- and calcium-dependent proteolytic enzymes. In humans, over 24 MMPs, either membrane-anchored or secreted forms, were found^7^. They are categorized into several types, including collagenases, gelatinases, stromelysins, matrilysins, membrane-type MMPs, and others^7^. MMP1 is a secreted protein belonging to the collagenase group. One key feature of collagenases is their ability to cleave interstitial collagens and a number of other extracellular matrix (ECM) and non-ECM molecules^9^. MMP1 specifically degrades fibroblast growth factor binding protein, insulin-like growth factor binding proteins 2,3,5, and transforming growth factor-β binding protein and release those proteins^10^. During carcinogenesis, MMPs can mediate metastasis and affect the initiation and growth of tumors through the loss of cell adhesion, deregulation of cell division, and evasion of apoptosis^7^.

In a recent review article about the role of MMPs in esophageal cancer, it was found that tissue immunostaining or serum levels of gelatinases (MMP2 and MMP9) had diagnostic value with regard to the development and progression of esophageal cancer^11^. Additionally, overexpression of MMP1 has been found in a variety of cancer tissue specimens, including ESCC^11^,^12^,^13^,^14^,^15^,^16^,^17^ and esophageal adenocarcinoma^11^,^17^,^18^,^19^, stained by immunostaining. Although previous studies have examined the feasibility of using plasma MMP1 as diagnostic or prognostic markers for lung cancers^20^, prostate cancer^21^, thyroid cancer^22^ and hepatocellular carcinoma^23^, it has not been investigated as such in esophageal cancer. To find out noninvasive biomarkers capable of detecting the presence and prognosis of ESCC, we first perused our own microarray data and microarray data obtained from public websites to search for the candidate secreted genes^6^,^24^,^25^. We then measured the protein expression of MMP1 in plasma in another set of ESCC patients and healthy controls to test our hypothesis.

## Results

### Identification of candidate genes for clinical application

**Figure 1:**
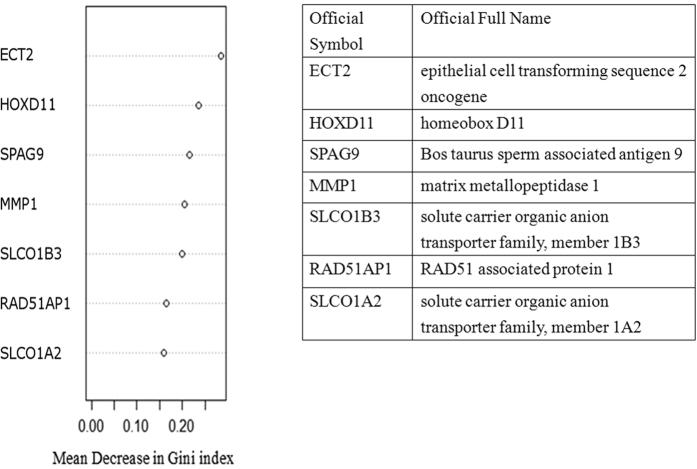
Identification of differentially expressed genes from microarray of 17 paired esophageal tissues. Seven genes were identified to have a fold-change >1.5 and a p-value < 0.005. Their importance on discriminating ESCC from normal tissues was evaluated by the mean decrease in Gini index (MDG) obtained from the Random Forests algorithm. Among the 7 genes, MMP1 ranked number four according to the MDG value.

We used a two-step method to identify significant differentially expressed genes from the microarray results of the 17 paired esophageal tissues. First, 7 genes (ECT2, HOXD11, SPAG9, MMP1, SLCO1B3, RAD51AP1, and SLCO1A2) were identified as having a fold-change above 1.5 and a p-value less than 0.005. The MDG values obtained from the Random Forests algorithm indicated the importance of the seven genes on discriminating ESCC from normal tissues (Fig. 1). Second, we reviewed the literature to select which of the 7 genes encode secreted or membrane proteins known to be involved in carcinogenesis. Because MMP1 was ranked number four in importance based on MDG value and was the only secreted protein measurable in both tissue and blood, we focused on MMP1 in our subsequent studies. Overexpression of MMP1 was detected in all patients in the microarray analysis. The minimum, mean, and maximum values of tumor/normal (T/N) MMP1 expression ratios were 3.23, 213.39, and 1148.64 respectively. The intensity of MMP1 expression and the T/N ratios of each pair of tissues are listed in Supplementary Table 1.

### Validation: Comparison of MMP1 expressions found in our microarray data with those published for Chinese ESCC patients and MMP1 protein expression in two ESCC tissues

Ninety-eight percent of the patients (52/53) in the GSE23400 dataset had higher expression of MMP1 in tumor tissues than normal tissues. In the only patient that did not have overexpression of MMP1, the T/N MMP1 expression ratio was 0.86. The other 52 patients had minimum, mean, and maximum T/N ratios of 1.27, 46.67, and 187.7 respectively. The seventeen patients in the GSE20347 dataset had minimum, mean, and maximum values of T/N ratios of 1.51, 90.05, and 466.1064, respectively (Supplementary Fig. 1). MMP1 was overexpressed in all ESCC tissues. In our study, we also found that the Immunostaining of MMP1 was stronger in tumor cells than in adjacent normal parts in the two ESCC patients (Supplementary Fig. 2).

### Plasma MMP1 levels and the detection of ESCC status

Plasma MMP1 was significantly higher in ESCC patients than that in controls (means ± standard deviations 8.7 ± 7.5 vs. 6.7 ± 4.9 ng/mL; p < 0.0001) (Table 1). While similar in gender, the control subjects were younger than the patients (45.5 ± 10.6 vs. 57.6 ± 12.1; p = 0.043). There was a significant association between ESCC and lower educational levels, consumption of tobacco, alcoholic beverages, and betel quids (all p-values < 0.0001; Table 1).

**Figure 2:**
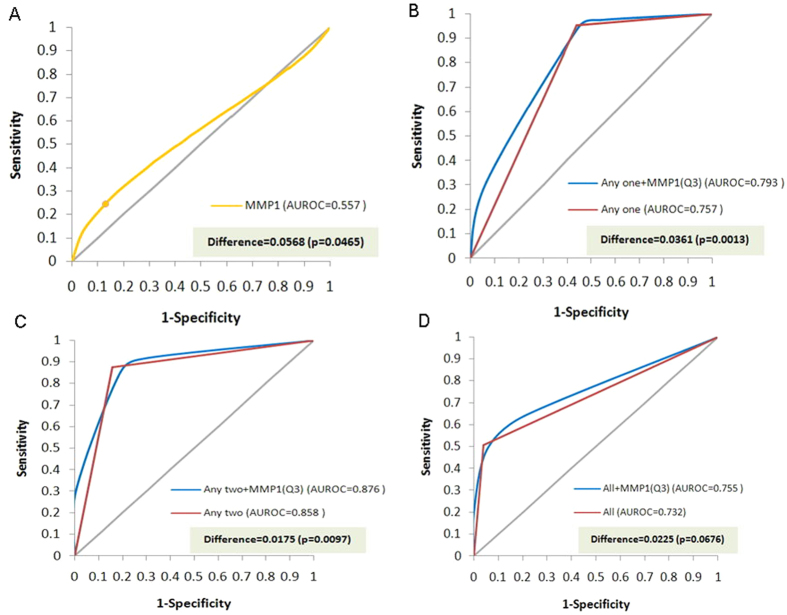
The nonparametric area under the receiver operating characteristic curve (AUROC) of plasma MMP1 with and without the three major risk factors of ESCC (alcohol drinking, smoking and betel quid chewing). (**A**) The AUROC of MMP1 alone was 0.557; the optimal cut-off value was very close to the highest quartile of all study subjects (9.67 ng/mL). (**B**) Among subjects with any one of the risk factors, adding plasma MMP1 significantly increased the AUROC by 0.0361 (p = 0.0013). (**C**) Among subjects with any two of the risk factors, adding plasma MMP1 significantly increased the AUROC by 0.0175 (p = 0.0097). (**D**) Among subjects with all three risk factors, adding plasma MMP1 increased the AUROC by 0.0225 (p = 0.0676).

The AUROC analysis for plasma MMP1 (Fig. 2A) identified the highest quartile of all subjects to be the optimal cut-off level (9.67 ng/mL). Compared with those with plasma MMP1 ≤ 9.67 ng/mL, subjects with higher levels had 9.0 times the risk of ESCC after adjustment for other covariates (AOR = 9.0, 95% CI = 2.2–36.0, p = 0.0019) (Table 2). Considering the effects of using different substances and MMP1 level together as predictors, we found heavy smokers (more than 20 pack-years) who had MMP1 levels >9.67 ng/mL to have 61.4 times the risk of ESCC (AOR = 61.4, 95% CI = 10.7–356.7) compared with non-smokers with lower MMP1 levels. Likewise, heavy drinkers (>20 drink-years) with higher MMP1 were also at much higher risk (AOR = 31.0, 95% CI = 6.1–161.6) compared to non-drinkers with lower plasma MMP1 (Table 2). ESCC risk tended to increase along with increases in the consumption of cigarettes or alcohol and MMP1 level.

**Figure 3:**
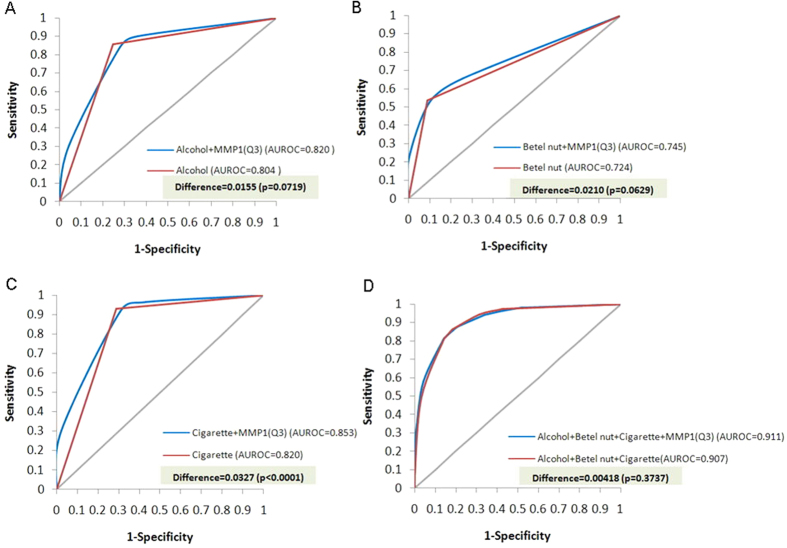
Comparison of the AUROC before and after adding plasma MMP1 to each risk factor of ESCC (alcohol drinking, smoking and betel quid chewing). (**A**) There was a borderline significant difference of AUROC after adding plasma MMP1 to alcohol (difference = 0.0155, p = 0.0719). (**B**) There was a borderline significant difference of AUROC after adding plasma MMP1 to betel nut (difference = 0.0210, p = 0.0629). (**C**) adding plasma MMP1 to smoking habit significantly increased the AUROC by 0.0327 (p < 0.0001). (**D**) After considering all three risk factors, adding plasma MMP1 did not improve discrimination.

In the subgroup analyses of AUROC, the adding of plasma MMP1 (dichotomized by 9.67 ng/mL) to different uses of substances (smoke, alcohol or areca quid) made possible significantly better detection rate of ESCC in those who consumed any one or two substances (Fig. 2B, AUROC = 0.793 vs. 0.757, difference = 0.0361, p = 0.0013 and Fig. 2C, AUROC = 0.876 vs. 0.858, difference = 0.0175, p = 0.0097). However, the contribution of plasma MMP1 to better detection was borderline among subjects who consumed all three substances regularly (Fig. 2D, difference = 0.0255, p = 0.0676). Further analysis revealed the detection ability of plasma MMP1 was the most significant among smokers (Fig. 3C difference = 0.0327, p < 0.0001) but borderline among alcohol drinkers (Fig. 3A difference = 0.0155, p = 0.0719) and betel chewers (Fig. 3A difference = 0.0210, p = 0.0629).

Among the 137 study subjects (95 ESCC patients and 42 controls) with available information of two gene polymorphisms (*ADH1B* and *ALDH2*), we found that plasma MMP1 was significantly associated with the presence of ESCC after adjusting for the covariates including *ADH1B* and *ALDH2* (Supplementary Table 2). Other variables, including year of education, cigarette smoking, alcohol consumption, and *ALDH2*, were also significantly associated with the presence of ESCC, although the sample size of this subgroup was relatively small.

### Plasma MMP1 levels and the survival of ESCC patients

**Figure 4:**
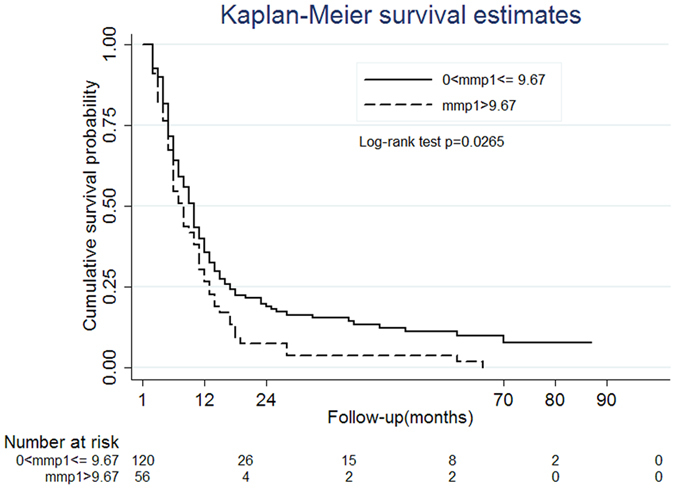
Kaplan-Meier survival curve of ESCC patients (N = 176) dichotomized by the cut-off level of plasma MMP1 (9.67 ng/mL). Patients with plasma MMP1 > 9.67 ng/mL had significantly shorter survival compared to those with lower MMP1 levels (p = 0.0265). Thirty-four patients were excluded for survival analysis because they died (N = 15) or lost follow-up (N = 19) within 1 month of cancer diagnosis.

Plasma MMP1 was not correlated with the clinical stage of ESCC, including T, N, M and overall ESCC stage (Supplementary Table 3). Among the 210 ESCC patients, 176 survived more than one month after the diagnosis of ESCC. Kaplan–Meier plot dichotomized by the cut-off level of MMP1 (9.67 ng/mL) showed the survival periods were statistically shorter in the high MMP1 group (p = 0.0265; Fig. 4). Compared to those with MMP1 ≤ 9.67 ng/mL, ESCC patients with MMP1 > 9.67 ng/mL had a 48% increase in the risk of ESCC death (adjusted hazard ratio = 1.48; 95% CI = 1.04–2.10) after adjusted for other covariates (Table 3).

## Discussion

This study found a consistent overexpression of MMP1 in ESCC tissues. Because *MMP1* can function as an oncogene and free MMP1 proteins can be released into blood from tumor cells, MMP1 could possibly be used as a non-invasive biomarker of ESCC. This consistent overexpression was found in our perusal of two public available microarray databases. Studying a set of plasma samples in ESCC patients and their controls, we found a significant association between high plasma MMP1 and ESCC. Although the diagnostic accuracy of plasma MMP1 alone was not very high, by adding this marker to substance use predictors we found a significant increase in the detection of ESCC in subjects who consumed any one or two of the three substances (alcoholic beverage, cigarette or betel quids). To best of our knowledge, this study is the first to support the value in diagnosis and outcome prediction of plasma MMP1 in ESCC. This study also showed dose-dependent interaction between substance use and plasma MMP1 level and the presence of ESCC. The borderline predictive significance when including MMP1 into our analysis of those consuming all three substances may partly be due to fewer case numbers (N = 106 and 8 for patients and controls, respectively).

MMPs are multifunctional enzymes that play complicated and sometimes opposing roles in several diseases, including cancer^7^. Increased MMP1 expression and accelerated ECM breakdown are observed in a various conditions, including inflammation, wound healing, chronic degenerative disease and cancers^7^. In cancer tissues, the expression of many types of MMPs is induced both in the cancer cells and in the surrounding stroma. These enzymes can affect many of the key processes involved in the tumorigenesis and progression of cancer^8^. MMP1 is one of the collagenases expressed in several cell types such as macrophages, epithelial cells and different kinds of cancers^26^. Huntington *et al*. found that activation of Ras/Raf/MEK/ERK pathway may be the driving force behind MMP1 overexpression in cancers^27^. MMP1 promotes tumor progression not only through ECM degradation of native fibrillar types I-III, and V collagens but also through regulation of the function of biologically active molecules by releasing them from ECM stores^10^.

Many agents, including pro-inflammatory cytokines and environmental factors such as cigarette smoking can stimulate the expression of MMP1. Kim *et al*. demonstrated an increased MMP1 mRNA and protein production by adding cigarette smoke extract to human pulmonary cells through activating ERK 1/2 pathway^28^. The association between single nucleotide polymorphisms (SNPs) of MMP1 and lung cancer risk was strongly increased among heavy smokers^29^. In this study, adding plasma MMP1 to smokers significantly increase the detection of ESCC. Such clinical observations may stem from the easier penetration of carcinogens due to looser cell adhesion after increased MMP1 (collagenase) expression, stimulated by cigarette smoking. However, it is not clear whether alcohol and betel quid directly influences MMP1 expression.

Elevated expression of MMP1 in the tumor tissues has been associated with the development or prognosis of a variety of cancers, including esophageal cancer^11^,^12^,^13^,^14^,^15^,^16^,^17^,^18^,^19^. The reported MMP1 protein expression in ESCC tissues has ranged from none to 72.9%. This wide variation, however, may be due to differences in case numbers, race and definition of protein upregulation. Two studies of Chinese populations have found 63–72.9% of the ESCC tumors they studied to be positively stained for MMP1^13^,^16^. It has been suggested that elevated MMP1 protein in tumors correlates with advanced diseases and poor prognosis^14^,^15^,^16^,^17^. However, one study in China (N = 208) did not find a significant association between tumor MMP1 expression and cancer stage or outcome^13^. The present study supported that plasma MMP1 could be an independent survival predictor for ESCC. A recent report indicated that MMP1 facilitated ESCC tumor growth and spread both *in vitro* and *in vivo*^30^. Their clinical data also showed high MMP1 expression in ESCC tissues was significantly related to shorter survival; the activation of the PI3K/AKT pathway played an important role^30^.

This study has several limitations. First, all patients were Taiwanese and very few of them were female, and thus care should be taken when generalizing our results to other populations. In addition, in the subgroup analysis such as Supplementary Table 2, because of small sample size, the ORs and 95% CIs of plasma MMP1 were instable and wide. Second, ESCC patients in this study were slightly older than their controls, though we controlled for this variable in our multivariable analyses. Third, only one measurement of MMP1 expression was available in this study. The future study should collect a serial measurement of MMP1 along with treatment (or cancer relapse) to further confirm its role as a tumor marker. Finally, this was a cross-sectional study, and no causal relationship of MMP1 expression with the development of ESCC could be established.

In conclusion, plasma MMP1 might be used as a non-invasive protein biomarker to assist in the detection of ESCC among subjects who consume alcoholic beverage, cigarette or betel quids. Further prospective cohort studies are necessary to investigate the possibility of using plasma MMP1 for selecting members of high-risk subpopulations for endoscopic surveillance to detect early ESCC before the development of phenotypic symptoms.

## Methods

### Microarray analysis of 17-paired human ESCC tumor and normal tissues

For microarray analysis, the tumor and adjacent normal tissues were obtained from 17 male ESCC patients who had received total esophagectomies without previous cancer treatment at Kaohsiung Medical University Hospital (KMUH). The detailed information about patient characteristics and microarray methods has been described previously^6^. Briefly, total RNA from each pair was isolated for the preparation of cDNA by reverse transcription. cDNA was then assayed by the Human Whole Genome OneArray (HOA v4.3, Phalanx Biotech Group, Hsinchu, Taiwan) containing 28,703 probes corresponding to the annotated genes in Unigene v175 and RefSeq database. The quality of each array in the entire experiment was evaluated by three steps: basic, reproducible and diagram. After the arrays had passed all three steps, the raw intensity of spots was log−2 transformed for subsequent analysis. Global Lowess normalizations were performed within repeated arrays of the same sample and between the samples to adjust for the systematic variation of experiments and dye effects. Spot was included for further analysis when it was “present” in at least one of the qualified arrays. The raw data has been uploaded to the Gene Expression Omnibus (GEO) database^31^.

### Identification of candidate genes that might be used to detect the presence of ESCC

The Random Forests classifier is capable of evaluating feature importance using out-of-bag (OOB) data^32^. Briefly, two-thirds and another one-third of data (OOB data) were used to build the classifier and evaluate the performance of that classifier. The importance for each gene was calculated by measuring the decrease of prediction performance of the permutated OOB data. In this study, Gini index was used as a measurement of prediction performance. The Gini index, a measure of impurity, represents the ability of a potential split for separating the samples of two classes and can be defined as 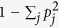, where *p*_*j*_ denotes the estimated class probabilities for a node and class *j* = 1, …, *J*. Generally, a gene with a large mean decrease in Gini index (MDG) is more important than a gene with a small MDG. Random Forests has been extensively used to rank variables, i.e. genes. The parameters of the numbers of trees and variables were set to 100 and 3, respectively.

### Validation of the array results by comparison of tissue MMP1 expressions in other microarray analyses of Chinese populations and MMP1 protein expression in two ESCC patients

To compare the expression of MMP1 in other studies, we retrieved two publicly available sets of microarray data (GSE23400^24^ and GSE20347^25^) from the Gene Expression Omnibus (GEO) database. GSE23400 and GSE 20347 consist of gene expression data from 53 and 17 Chinese ESCC patients, respectively. The MMP1 levels in tumors and corresponding normal specimens were compared and T/N ratios were calculated.

Immunohistochemistry (IHC) study was performed on the formalin-fixed paraffin-embedded ESCC tissues according to the manufacturer’s instruction using anti-MMP1 antibody (Merck; MAB-3307; 1:300 dilution) and anti-mouse/rabbit secondary antibody conjugated with HRP (ChemMate DAKO EnVision Detection Kit, Code: K5007, DAKO).

### Study population for MMP1 protein expression in plasma

Our study subjects consisted of patients with incident, pathologically-proven ESCC treated at KMUH and Kaohsiung Veterans General Hospital, two medical centers in Kaohsiung, Taiwan, between 2000 and 2008. Blood samples were obtained before any cancer treatment. Clinical information was obtained by reviewing the patients’ medical charts. Details regarding recruitment, cancer staging and principles of treatment have been described previously^4^. The controls were healthy subjects recruited from the Department of Preventive Medicine in the two hospitals during the same period that patients were recruited. They were recruited during a health checkup and were proven to be cancer-free after a series of exams including chest x-ray, abdominal/pelvic echo, upper endoscopy and colonoscopy. All of the participants were interviewed to collect demographic and lifestyle information using a standard questionnaire^4^. The plasma specimens were stored in a −80 °C freezer until analyses. This study was approved by the Institutional Review Boards of KMUH (KMUH-IRB-960420); written informed consent was obtained from all participants. All clinical investigations were conducted in accordance to the principles expressed in the Declaration of Helsinki.

### Plasma MMP1 measured by enzyme-linked immunosorbent assay (ELISA)

During the years 2000 and 2008, we collected 407 archived plasma specimens (210 case patients and 197 controls) which were stored at −80 °C until analysis. Plasma MMP1 protein levels were measured by ELISA according to the manufacturer’s instructions (R&D Systems, Inc., Minneapolis, MN, USA). A monoclonal antibody specific for MMP1 was precoated onto a microplate. Standards and samples were pipetted into the wells, and MMP1 was bound by the immobilized antibody. After washing away unbound substances, an enzyme-linked monoclonal antibody specific for MMP1 was added to the wells. Following a wash to remove unbound antibody-enzyme reagent, a substrate solution was added to the wells and color developed in proportion to the amount of MMP1 bound in the initial step. The intensity of the color development was measured using a microplate reader at 450 nm with a wavelength correction at 540 nm. Each assay was repeated two times. One technician (HS Lin), who was blinded to the clinical staging of ESCC patients, performed the measurements.

### Statistical Analysis

The differences of demographic characteristics, substance use and plasma MMP1 levels between patients and controls were analyzed by *t-*statistics for continuous variables or by χ^2^ for categorical variables. Because the cutoff value of MMP1 with optimal discriminatory ability, defined as the threshold yielding the maximum Youden index (J) calculated using the sum of sensitivity and specificity minus one, was close to the upper quartile of MMP1, we chose the top quartile as the cutoff point for subsequent analyses. Multivariate logistic regression was used to examine the association between plasma MMP1 concentrations alone or in combination with substances used and the presence of ESCC after adjusting for other covariates, including age, gender, educational levels and/or consumption of tobacco, alcohol or betel quid. The amount of substances consumed was divided into three groups according to the accumulated dose. For cigarette and betel quid, those groups were non-user, users who had consumed 1–20 pack-years, and those who had consumed >20 pack-years. Smoking or betel quid chewing pack-years were calculated by multiplying the number of cigarette/betel quid packs consumed per day by the number of years. One pack of betel-quid contains 10 betel quids. One alcohol drink was equivalent to a can of beer containing 17.5 g of alcohol.

In order to test whether plasma MMP1 increased the detection of ESCC among subjects with different substance use habits, we quantified the discrimination ability of the model by calculating the concordance statistic, which was identical to the nonparametric area under the receiver operating characteristic curve (AUROC). AUROC were plotted with sensitivity and 1-specificity along the vertical and horizontal axes, respectively. Substantially improved predictions were tested by evaluating whether the AUROC difference equaled zero in the nested models.

Previous studies, including ours, have found that functional polymorphisms of alcohol dehydrogenase (*ADH1B*) and aldehyde dehydrogenase (*ALDH2*) genes, located on chromosome 4q22 and 12q24, respectively, are highly associated with the risk of ESCC^33^,^34^. One amino acid change from arginine (CGC) (ADH1B*1) to histidine (CAC) (ADH1B*2) was noted in *ADH1B* gene at codon 47 of exon 3 and from glutamic acid (ALDH2*1) to lysine (ALDH2*2) was noted in *ALDH2* at codon 487 of exon 12. Thus, in the subgroup analysis with available information about these two gene polymorphisms, we examined the relationship between plasma MMP1 and the risk of ESCC after the further adjustment of these two gene polymorphisms.

To examine whether plasma MMP1 predicted the survival of ESCC patients, we used Kaplan–Meier analysis and log-rank testing in crude analysis and Cox proportional hazards modeling with computing hazard ratios (HRs) and 95% CIs in multivariable analysis. Each participant accumulated person-time beginning from the ESCC diagnostic date and ending on the date of ESCC death or the end of this study in January 2016. We excluded the ESCC patients who died within one month (N = 15) or lost follow-up (N = 19) after initial cancer diagnosis because they usually had very poor performance, severe infection or refused cancer treatment; all affected plasma MMP1 levels or survival. The covariates in Cox regression included the above-mentioned variables in logistic regression plus clinical staging. The data were analyzed using the SAS statistical package. A p-value < 0.05 was considered significant.

## Additional Information

**How to cite this article**: Chen, Y.-K. *et al*. Plasma matrix metalloproteinase 1 improves the detection and survival prediction of esophageal squamous cell carcinoma. *Sci. Rep.* **6**, 30057; doi: 10.1038/srep30057 (2016).

## Supplementary Material

Supplementary Information

## Figures and Tables

**Table 1:** Distribution of plasma MMP1 levels and demographic characteristics in ESCC patients and controls.

Variables	Controls n = 197	Patients n = 210	*p*
**Mean ± SD or N (%)**			
**MMP1**	**6.7 ± 4.9**	**8.7 ± 7.5**	**<0.0001**
Age (years)	45.5 ± 10.6	57.6 ± 12.1	0.043
Gender			
male	195 (99)	205 (98)	0.304
female	2 (1)	5 (2)	
Year of education (years)			
<9	44 (22)	142 (68)	<0.0001
9–12	32 (16)	40 (19)	
>12	118 (60)	15 (7)	
missing	3 (2)	13 (6)	
Cigarette smoking			
No	141 (72)	15 (7)	<0.0001
Yes	56 (28)	188 (90)	
missing	0 (0)	7 (3)	
Alcohol consumption			
No	146 (74)	31 (15)	<0.0001
Yes	51 (26)	170 (81)	
missing	0 (0)	9 (4)	
Betel quid chewing			<0.0001
No	183 (93)	97 (46)	
Yes	14 (7)	106 (51)	
missing	0 (0)	7 (3)	

Abbreviation: ESCC: esophageal squamous cell carcinoma; SD: standard deviation.

**Table 2:** Association between plasma MMP1 concentrations and ESCC risk.

MMP1 (ng/mL)	Control (n = 197) No. (%)	Case (n = 210) No. (%)	Crude OR (95% CI)^1^	Adjusted OR^1^ (95% CI)
Quartile				
<3.69	46 (23)	50 (24)	1.0	1.0
3.69–5.84	58 (29)	49 (23)	0.8 (0.4–1.4)	1.0 (0.4–2.8)
5.85–9.67	61 (31)	46 (22)	0.7 (0.4–1.2)	1.1 (0.4–3.3)
>9.67	32 (16)	65 (31)	1.9 (1.0–3.3)*	9.3 (2.1–41.3)†
Dichotomy				
≤9.67	165 (84)	145 (69)	1.0	1.0
>9.67	32 (16)	65 (31)	2.3 (1.4–3.7)#	9.0 (2.2–36.0)‡
Cigarette smoking (pack-year)/MMP1 (ng/mL)				
No/≤9.67	113 (58)	10 (5)		1.0
No/>9.67	28 (14)	5 (2)		5.7 (1.2–26.3)
1–20/≤9.67	15 (8)	27 (13)		15.1 (4.2–54.8)
1–20/>9.67	2 (1)	14 (7)		27.0 (3.4–215.8)
>20/≤9.67	36 (17)	108 (51)		15.5 (5.3–45.2)
>20/>9.67	3 (2)	46 (22)		61.4 (10.7–356.7)
Betel-quid chewing (pack-year)/MMP1 (ng/mL)				
No/≤9.67	150 (76)	81 (39)		1.0
No/>9.67	33 (17)	23 (11)		2.8 (0.9–8.4)
1–20/≤9.67	7 (4)	21 (10)		1.9 (0.6–6.0)
1–20/>9.67	0 (0)	7 (3)		—
>20/≤9.67	7 (4)	43 (20)		3.1 (0.9–10.1)
>20/>9.67	0 (0)	35 (17)		—
Alcohol drinking (drink-year)/MMP1 (ng/mL)				
No/≤9.67	125 (63)	32 (15)		1.0
No/>9.67	21 (11)	8 (4)		5.3 (1.0–26.9)
1–20/≤9.67	17 (9)	23 (11)		5.6 (1.7–18.7)
1–20/>9.67	8 (4)	9 (4)		15.9 (2.8–91.8)
>20/≤9.67	22 (11)	90 (43)		7.6 (3.0–19.1)
>20/>9.67	4 (2)	48 (23)		31.0 (6.0–161.6)

Abbreviation: CI: confidence interval; ESCC: esophageal squamous cell carcinoma; OR: odds ratio.

*p = 0.0355; ^#^p = 0.0006; ^†^p = 0.0035; ^‡^p = 0.0019.

One pack of betel-quid contains 10 betel-quids; one alcohol drink equals to a beer can containing 17.5 g of alcohol.

^1^Adjusted for age, sex, educational levels, smoking, alcohol and betel chewing.

**Table 3:** Relationship of plasma MMP1 with the survival of ESCC patients in Cox regression models (N = 176).

Variables	Crude HR	95% CI		Adjusted HR	95% CI	
MMP1 (ng/mL)						
≤9.67	1			1		
>9.67	1.43	1.03	1.99	1.48	1.04	2.10
Age (years)	1.01	1.00	1.02	1.02	1.01	1.04
Gender						
male	1			1		
female	4.18	1.30	13.42	3.90	1.04	14.64
Year of education (years)						
<9	1			1		
9–12	1.01	0.69	1.47	1.27	0.85	1.92
>12	1.08	0.61	1.91	1.19	0.63	2.23
missing	0.86	0.40	1.84	0.91	0.36	2.25
Cigarette smoking						
No	1			1		
Yes	0.73	0.43	1.22	0.77	0.38	1.54
missing	1.41	0.45	4.43	2.17	0.26	17.84
Alcohol consumption						
No	1			1		
Yes	1.24	0.82	1.86	1.31	0.77	2.23
missing	0.74	0.28	2.01	0.48	0.11	2.17
Betel quid chewing						
No	1			1		
Yes	0.77	0.57	1.05	0.74	0.51	1.07
missing	0.90	0.22	3.65	0.41	0.02	8.40
Stage						
Stage I, II	1			1		
Stage III, IV	1.64	1.16	2.32	1.66	1.14	2.41
missing	4.33	0.59	31.93	8.64	1.02	73.46

Abbreviation: CI: confidence interval; ESCC: esophageal squamous cell carcinoma; HR: hazard ratio.

Adjusted for all variables listed in this Table.
