# Supplementary material for: Plasma matrix metalloproteinase 1 improves the detection and survival prediction of esophageal squamous cell carcinoma
Source: Sci Rep. 2016 Jul 20;6:30057. doi: 10.1038/srep30057 (PMC4951749; doi:10.1038/srep30057)
Supplement: Supplementary Information [file srep30057-s1.pdf]

Plasma matrix metalloproteinase 1 improves the detection and survival prediction of esophageal squamous cell carcinoma

Yu-Kuei Chen[1], Chun-Wei Tung[2], Jui-Ying Lee[3], Yi-Chun Hung[4], Chien-Hung Lee[5], Shah-Hwa Chou[3], Hung-Shun Lin[6], Ming-Tsang Wu[4,5], I-Chen Wu[7,8+]

[1]Department of Food Science and Nutrition, Meiho University, Pingtung, Taiwan

[2]School of Pharmacy, Kaohsiung Medical University, Kaohsiung, Taiwan

[3]Division of Chest Surgery, Department of Surgery, Kaohsiung Medical University Hospital, Kaohsiung, Taiwan

[4]Research Center for Environmental Medicine, Kaohsiung Medical University, Kaohsiung, Taiwan

[5]Department of Public Health, Kaohsiung Medical University, Kaohsiung, Taiwan

[6]Department of Laboratory Medicine & Department of Research, Education & Training, Kaohsiung Municipal Hsiao-Kang Hospital, Kaohsiung Medical University, Kaohsiung, Taiwan

[7]Division of Gastroenterology, Department of Internal Medicine, Kaohsiung Medical University Hospital, Kaohsiung, Taiwan

[8]Faculty of Medicine, Department of Medicine, College of Medicine, Kaohsiung Medical University, Kaohsiung, Taiwan

sTable 1. MMP1 expression in 17 pairs of esophageal specimens

Case No.	1		2		3		4		5		6		7		8		9		10		11		12		13		14		15		16		17	
Part	N	T	N	T	N	T	N	T	N	T	N	T	N	T	N	T	N	T	N	T	N	T	N	T	N	T	N	T	N	T	N	T	N	T
Intensity	187	616	185	40897	93	10365	79	42772	92	45424	65	24060	82	455	62	4124	818	2644	45	271	142	11557	388	25396	94	40937	38	713	48	1551	42	48243	31	717
T/ N ratio		**3**		**221**		**111**		**541**		**494**		**370**		**6**		**67**		**3**		**6**		**81**		**65**		**436**		**19**		**32**		**1149**		**23**

N: Normal T: Tumor

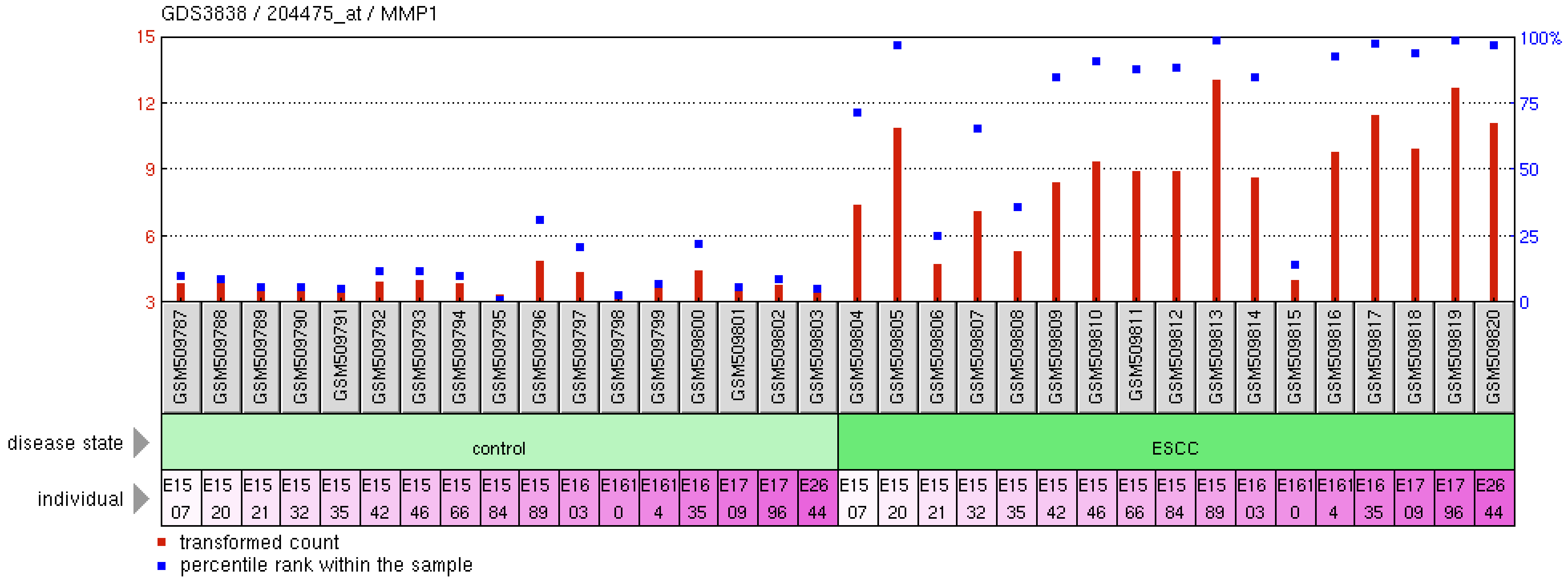

sFigure. 1. The ratio of MMP1 expression in tumor/normal parts from 17-paired ESCC samples (Data from Hu et al., 2010)

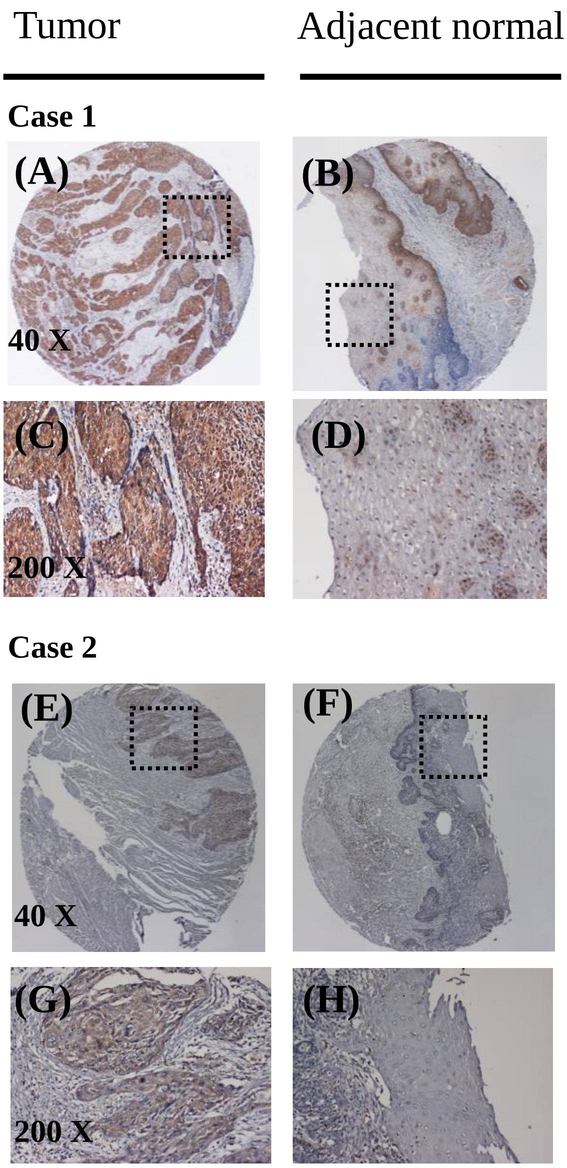

sFigure 2. MMP1 expression in ESCC and adjacent normal tissues. (A) and (B) MMP1 expression in case 1 ×40X, (C) and (D) MMP1 expression in case 1 ×200X, (E) and (F) MMP1 expression in case 2 ×40X, (G) and (H) MMP1 expression in case 2 ×200X. Immunostaining of MMP1 was stronger in tumor cells than in adjacent normal parts.

sTable 2. Association between plasma MMP1 and ESCC risk in ESCC patients and controls with available information of genotypes (N = 137).

	**Patients**	**Controls**		
	**N = 95**	**N = 42**	**Crude OR (95% CI)**	**Adjusted OR (95% CI)**
	**N (%) or Mean ± SD**			
**MMP1 (ng/mL)**				
**≤ 9.67**	**64 (67.37)**	**39 (92.86)**	**1**	**1**
**> 9.67**	**31 (32.63)**	**3 (7.14)**	**6.30 (1.80-21.98)**	**40.60 (1.66-990.53)**
Age (years)	58.33±13.11	56.74±10.59	1.01 (0.98-1.04)	1.05 (0.99-1.12)
Year of education (years)				
<9	70 (73.68)	11 (26.19)	1	1
9-12	14 (14.74)	5 (11.90)	1.28 (0.43-3.81)	0.54 (0.07-4.02)
>12	6 (6.32)	23 (54.76)	0.56 (0.02-0.16)	0.08 (0.01-0.45)
missing	5 (5.26)	3 (7.14)	0.72 (0.16-3.17)	0.27 (0.02-3.76)
Cigarette smoking				
No	4 (4.21)	24 (57.14)	1	1
Yes	91 (95.79)	18 (42.86)	30.33 (9.39-98.03)	18.54 (2.83-121.50)
Alcohol consumption				
No	11 (11.58)	31 (73.81)	1	1
Yes	84 (88.42)	11 (26.19)	21.52 (8.48-54.64)	11.26 (2.20-57.68)
Betel quid chewing				
No	44 (46.32)	38 (90.48)	1	1
Yes	51 (53.68)	4 (9.52)	11.01 (3.64-33.29)	4.86 (0.90-26.13)
ADH1B				
*2/*2	35 (36.84)	22 (52.38)	1	1
*1/*2 + *1/*1	60 (63.16)	20 (47.62)	1.89 (0.90-3.93)	0.72 (0.16-3.26)
ALDH2				
*1/*1	20 (21.05)	21 (50.00)	1	1
*1/*2 + *2/*/2	75 (78.95)	21 (50.00)	3.75 (1.72-8.18)	4.73 (1.00-22.41)

Abbreviation: *ADH1B*: alcohol dehydrogenase; *ALDH2*: aldehyde dehydrogenase; CI: confidence interval; ESCC: esophageal squamous cell carcinoma; OR: odds ratio; SD:standard deviation. Adjusted for all variables listed in this table.

sTable 3. Relationship between TNM stage and plasma MMP1 (dichotomized by the highest quartile level of all study subjects)

**MMP1 (ng/ mL)**<br>**Variables**	**≤9.67**<br>**n (%)**	**>9.67**<br>**n (%)**	***p* value**
T			
T1-T2	27 (66)	14 (34)	0.9
T3-T4	111 (71)	46 (29)	
missing	7 (58)	5 (42)	
N			0.63
N0	41 (67)	20 (33)	
N1	97 (71)	40 (29)	
missing	7 (58)	5 (42)	
M			0.36
No	107 (71)	44 (29)	
Yes	38 (64)	21 (36)	
Stage			0.64
Stage I, II	40 (67)	20 (33)	
Stage III, IV	100 (70)	43 (30)	
missing	5 (71)	2 (29)	
